# Ileal Transposition in Rats Reduces Energy Intake, Body Weight, and Body Fat Most Efficaciously When Ingesting a High-Protein Diet

**DOI:** 10.1007/s11695-020-04565-6

**Published:** 2020-04-27

**Authors:** Edit Somogyi, David Sigalet, Thomas E. Adrian, Csaba Nyakas, Christiaan W. Hoornenborg, André P. van Beek, Henry S. Koopmans, Gertjan van Dijk

**Affiliations:** 1 School of PhD Studies, University of Physical Education, Budapest, Hungary; 2 Department of Behavioral Neuroscience, Groningen Institute for Evolutionary Life Sciences (GELIFES), University of Groningen, Groningen, the Netherlands; 3 Faculty of Medicine, University of Calgary, Calgary, AB Canada; 4 Department of Basic Medical Sciences, Mohammed Bin Rashid University of Medicine and Health Sciences, Dubai, UAE; 5 Department of Morphology and Physiology, Faculty of Health Sciences, Semmelweis University, Budapest, Hungary; 6 Department of Endocrinology, University of Groningen, University Medical Center Groningen, Groningen, the Netherlands; 7 Department of Physiology and Biophysics, Faculty of Medicine, University of Calgary, Calgary, Canada

**Keywords:** Ileal transposition, Energy balance, Food intake, Energy efficiency, PYY

## Abstract

**Purpose:**

Ileal transposition (IT) allows exploration of hindgut effects of bariatric procedures in inducing weight loss and reducing adiposity. Here we investigated the role of dietary macronutrient content on IT effects in rats.

**Methods:**

Male Lewis rats consuming one of three isocaloric liquid diets enriched with fat (HF), carbohydrates (HC), or protein (HP) underwent IT or sham surgery. Body weight, energy intake, energy efficiency, body composition, and (meal-induced) changes in plasma GIP, GLP-1, PYY, neurotensin, and insulin levels were measured.

**Results:**

Following IT, HC intake remained highest leading to smallest weight loss among dietary groups. IT in HF rats caused high initial weight loss and profound hypophagia, but the rats caught up later, and finally had the highest body fat content among IT rats. HP diet most efficaciously supported IT-induced reduction in body weight and adiposity, but (as opposed to other diet groups) lean mass was also reduced. Energy efficiency decreased immediately after IT irrespective of diet, but normalized later. Energy intake alone explained variation in post-operative weight change by 80%. GLP-1, neurotensin, and PYY were upregulated by IT, particularly during (0–60 min) and following 17-h post-ingestive intake, with marginal diet effects. Thirty-day post-operative cumulative energy intake was negatively correlated to 17-h post-ingestive PYY levels, explaining 47% of its variation.

**Conclusion:**

Reduction in energy intake underlies IT-induced weight loss, with highest efficacy of the HP diet. PYY, GLP-1, and neurotensin levels are upregulated by IT, of which PYY may be most specifically related to reduced intake and weight loss after IT.

## Introduction

Overweight and obesity are among the greatest health problems facing the world today, because of its comorbidities [1–4] and the rise in associated healthcare costs [5]. Among different weight loss strategies currently available [6, 7], bariatric surgery emerged as the most effective, and ileal transposition (IT) is one of these surgeries [8, 9]. IT was first described by Koopmans et al. [8, 10] and is a procedure in which a lower part of the ileum is surgically transposed just below the common bile and pancreatic duct, which then presumably causes earlier- and over-stimulation of hormone-producing enteroendocrine cells [9, 11] of the transposed segment. IT is therefore an ideal non-restrictive model without malabsorption [12] to explore hindgut effects of bariatric procedures in inducing weight loss.

Chen et al. [12] showed that rats undergoing IT had a decreased preference for fat [12]. Advanced nutrient exposure to the transposed ileal segment could play a role in this phenomenon [13, 14]. Because of this finding, it would be expected that weight loss following IT would be relatively high in rats subjected to a high-fat diet. On the other hand, a high-protein diet has been shown to be more satiating than a high-fat or high-carbohydrate diet in both humans [15, 16] and rodents [17, 18], and enhances weight and fat loss [19–21]. Proteins [22] and, probably more potently, lipids [23–25] could strongly induce the ileal brake mechanism to provide optimal absorption of nutrients, and may induce satiety. Given these presumed effects of macronutrients on energy balance regulation and gut physiology, we investigated the effect of diet, either enriched in fat, carbohydrates, or protein in a rat model of IT surgery, on post-surgery weight change, energy intake, and energy efficiency, as well as on circulating gut hormone levels.

## Methods

Fifty-four male Lewis rats (mean weight 310 g) were housed individually in plastic cylindrical cages (diameter 33 cm, height 50 cm) with rat chow (Labdiet®, PROLAB RMH2500 Rodent diet, PMI Nutrition International, LLC, MO, USA) and water ad libitum under artificial lighting (6 a.m.–6 p.m.) at room temperature. This study was performed in male rats because of potential complicating effects of estrus cyclicity in female rats on energy balance regulation, which would require a more complex study design.

After 6 days of acclimatization, rats were separated into three weight-matched groups and were maintained on high-fat (HF, *n* = 18), high-carbohydrate (HC, *n* = 18), and high-protein (HP, *n* = 18) liquid diets, which they could freely ingest daily between 4 p.m. (i.e., 2 h before lights off) and 9 a.m. (i.e., 3 h after lights on) the next day. Diet jars were weighed at the beginning and at the end of feeding intervals, allowing assessment of daily energy intake (EI). Rats were weighed daily at 3.30 p.m., before diet presentation. After 8 days on the diets, rats were matched for body weight and body weight gain and divided into two surgical groups: ileal transposition (IT+, *n* = 9 per diet group) and control surgery (IT−, *n* = 9 per diet group). All applicable institutional and/or national guidelines for the care and use of animals were followed.

### Diets

Diets consisted of mixtures of Ensure Plus (Abbott Canada Saint-Laurent, Québec, Canada), Resource Beneprotein powder (Novartis Medical Nutrition, USA), 3) Intralipid 20% (Fresenius Kabi Clayton L.P., Clayton, NC) and Maltlevol liquid vitamin mix (Carter-Horner Corp Mississauga ON, Canada), and water (Table 1). This yielded three isocaloric diets of 4.184 kJ/g, with fat/carbohydrate/protein energy percentages of resp. 50/25/25 (HF diet), 25/50/25 (HC diet), or 25/25/50 (HP diet).

**Table 1:** Ingredients of high-fat (HF), high-carbohydrate (HC), and high-protein (HP) diets per kilogram

	Mass (g)	Fat (kJ)	Carbohydrate (kJ)	Protein (kJ)	Sum (kJ)
HF diet					
Ensure Plus	302.0	547.1	1066.6	278.0	1891.6
Beneprotein	53.1			761.9	761.9
Maltevol	8.0				
Intralipid (20%)	182.2	1530.6			1530.6
Water	454.7				
Energy (kJ)		2077.6	1066.6	1039.9	4184.1
Energy (%)		50	25	25	
HC diet					
Ensure Plus	587.6	1064.3	2074.9	540.9	3680.0
Beneprotein	35.1			504.0	504.0
Maltevol	7.8				
Intralipid (20%)					
Water	369.5				
Energy (kJ)		1064.3	2074.9	1044.8	4184.0
Energy (%)		25	50	25	
HP diet					
Ensure Plus	296.3	536.8	1046.5	272.8	1856.0
Beneprotein	128.2			1839.0	1839.0
Maltevol	9.4				
Intralipid (20%)	58.2	489.1			489.1
Water	507.8				
Energy (kJ)		1025.9	1046.5	2111.8	4184.1
Energy%		25	25	50	

### Ileal Transposition

After overnight fast, rats were anesthetized with ether, and the skin and muscle layer of the belly were cut at the midline exposing the abdomen. The small intestine was then transected at the level of (1) the duodenum 1 to 2 cm below the common bile and pancreatic duct, (2) the ileum at 10 cm from the ileocecal valve, and (3) the ileum 10 cm above this transection, creating an isolated 10-cm ileal segment. For the ileal transposition surgery, the 10-cm ileal segment was connected (using 6–0 Ethicon silk sutures) to the transected ends of the duodenum in the original direction of flow keeping its mesenteric blood supply and extrinsic innervation intact. The remaining ends of the ileum were sutured together, resulting in a gastrointestinal tract which had its original length without any excluded parts. For the control surgery, all transections were re-anastomosed in their original order, returning the intestine to its continuity. After surgery, rats returned to their home cages with a heating pillow underneath it, and they received Gentamicin (i.p. 37 μl/100 g body weight, 40 mg/ml, Sabex Inc. Boucherville QC) and butorphanol tartrate; i.p. 0.2 mg/100 g body weight, 10 mg/ml, Wyeth Canada Guelph, ON). Rats did not have access to any diet for 24 h after surgery, but water was freely available.

### Blood Sampling

About 5 weeks after IT+ or IT−, an indwelling catheter (Silastic tubing 0.025 i.d. × 0.047 o.d., Dow Corning Corp., Midland MI) with a Marlex mesh (Prolene Mesh, Ethicon) anchor located in the middle of the tube was inserted under ether anesthesia into the jugular vein. The other end of the tube was tunneled under the skin to the back of the neck. Between sampling, catheters were filled with a mixture of 8 g of polyvinylpyrrolidine (BDH Chemicals Ltd., Poole, UK), 10 ml of heparin (1000 units/ml), and 5 mg Enrofloxacin (Baytril, Bayer Inc., Etobicoke, ON) and gentamicin (37 μl/100 g body weight, 40 mg/ml) and sealed with a stainless steel wire hook. They were reopened on the third day after surgery and 3.6 ml blood was withdrawn (collected with 1.5 mg/ml EDTA) and centrifuged and plasma was stored at 4 °C in sterile vials.

A total of five samples were taken per animal at time points − 10 min (before feeding), and 15, 30, 60 min, and 17 h after feeding had started. There were two criteria for blood sampling: (1) When rats had ingested 5-ml meal within 4 min, which was determined from the animals’ short-term food intake during the first 33 days of the study; (2) the 17-h samples were taken, when the rats had eaten approximately the same amount of food as they did during the period between the 24th and 30th day of the study. Two blood samples of 1.8 ml were taken on the fourth day after reopening of the catheters and collected into syringes containing 0.08 ml (50 Kallikrein Inactivating Units [KIU]/ml) aprotonin (Trasylol proteinase inhibitor, Bayer, Germany) and 1.5 mg/ml EDTA. In between collection of samples, an equivalent amount of the rat’s own warmed serum was re-infused to replace drawn blood. After the two blood samples, the rats had food and water freely available for the rest of the night. The second pair of blood samples was withdrawn after a day without blood sampling and the final blood sample was collected after sacrifice. All blood samples were immediately transferred into plastic tubes and centrifuged, and plasma was stored at − 20 °C until analysis.

### Hormone Assays

Plasma samples were extracted individually on C-18, reverse phase Sep-Pak cartridges (Waters, Inc., Milford, MA), using multichannel syringe ram pumps (Harvard Instruments, Cambridge, MA). After washing, cartridges were eluted with 3 ml of 50% acetonitrile with 0.1% trifluoroacetic acid. Eluates were lyophilized and reconstituted in buffer consisting of 60 mM phosphate pH 7.4 with 0.1% Triton-X 100 and 0.1% bovine albumin. Extraction recovery was determined in each assay by adding 0, 5, or 20 fmol peptide/ml to 10 pooled rat plasma samples before extraction. Plasma hormone concentrations were measured simultaneously and using a previously standardized and established radioimmunoassays, specific and sensitive to rat gastrointestinal hormones such as peptide YY (PYY) [26], glucagon-like peptide 1-amide (GLP-1) [27], glucose-dependent insulinotropic polypeptide (GIP) [28], neurotensin (NT) [29], and insulin [30]. Labeled peptides were produced by conventional chloramine T oxidation. Mono-iodinated peptides were purified using high-resolution, reverse phase high-pressure liquid chromatography. Antisera were added at a dilution which binds approximately 50% of the 1–1.5 fmol of labeled peptide in the absence of non-labeled peptide. Assays were incubated for 7 days under equilibrium conditions. Free from bound hormone was separated by precipitation of the latter by addition of polyethylene glycol to give a final concentration of 12% PEG (mol wt 6000) and addition of 1 mg/tube of crude gamma-globulin to make a visible precipitate. Supernatants were removed using a vacuum line, and samples were counted on a 10-well auto gamma counter (model 1277, LKB/Pharmacia Inc., Chicago, IL) with data reduction accomplished by RIACALC software.

### Energy Efficiency Calculation

Energy efficiency is the ability of the rat to efficiently use the energy intake for the use of body (weight) gain over a period of time. We calculated energy efficiency by using the following equation [31]:

$$ \mathrm{Energy}\ \mathrm{efficiency}=\left\{\Delta \ \mathrm{Bodyweight}\ \left(\mathrm{kg}\right)\ \mathrm{per}\ \mathrm{day}/\mathrm{average}\ \mathrm{daily}\ \mathrm{energy}\ \mathrm{inatake}\ \left(\mathrm{kJ}\right)\right\} $$

### Body Composition Analysis

Rats were euthanized by decapitation under ether anesthesia between days 49 and 52 after surgery. The liver and kidneys were separated and weighed. Adipose tissue pads were dissected and weighed substantiating visceral and subcutaneous depots. The gastrointestinal tract (GI) was removed and weighed with content. The GI was divided in stomach, (upper and lower) duodenum, transposed segment and last 10 cm of ileum, total small intestine, cecum, and colon with content were weighed. The gastrocnemius muscle was dissected and weighed as a representative of lean muscle tissue.

### Statistical Analysis

For the body weight data, differences from baseline were calculated following surgery. Data on food intake, body weight, energy efficiency, hormone responses, and carcass analysis were analyzed with univariate ANOVA and Tukey’s pairwise multiple comparison with diet (HF, HC, HP) and surgery (IT−, IT+) as factors. The hormone data were presented as baseline (semi-fasted), as post-meal (0–60 min) incremental area under the curve (AUC), and as post-ingestive levels (i.e., 17 h after start of the blood sampling). The meal-induced gut hormone responses are shown in the Appendix (Figs. 4, 5, 6, 7, 8). Linear regression was performed to assess potential relations between surgery, diet, energy intake, body weight changes, and gut hormone levels. Data is presented as mean ± se and *p* values less than 0.05 were considered significant. Two IT− rats (HP and HC) and one IT+ rat (HF) were euthanized shortly after surgery because of poor recovery (leakage of the anastomosis), and one additional rat could not be used for the blood sampling, because of a clogged catheter (HC IT−). In two additional rats (one HC IT+, and one HP IT−), we did not have records of intake during the period of blood sampling.

## Results

### Body Weight

**Fig. 1:**
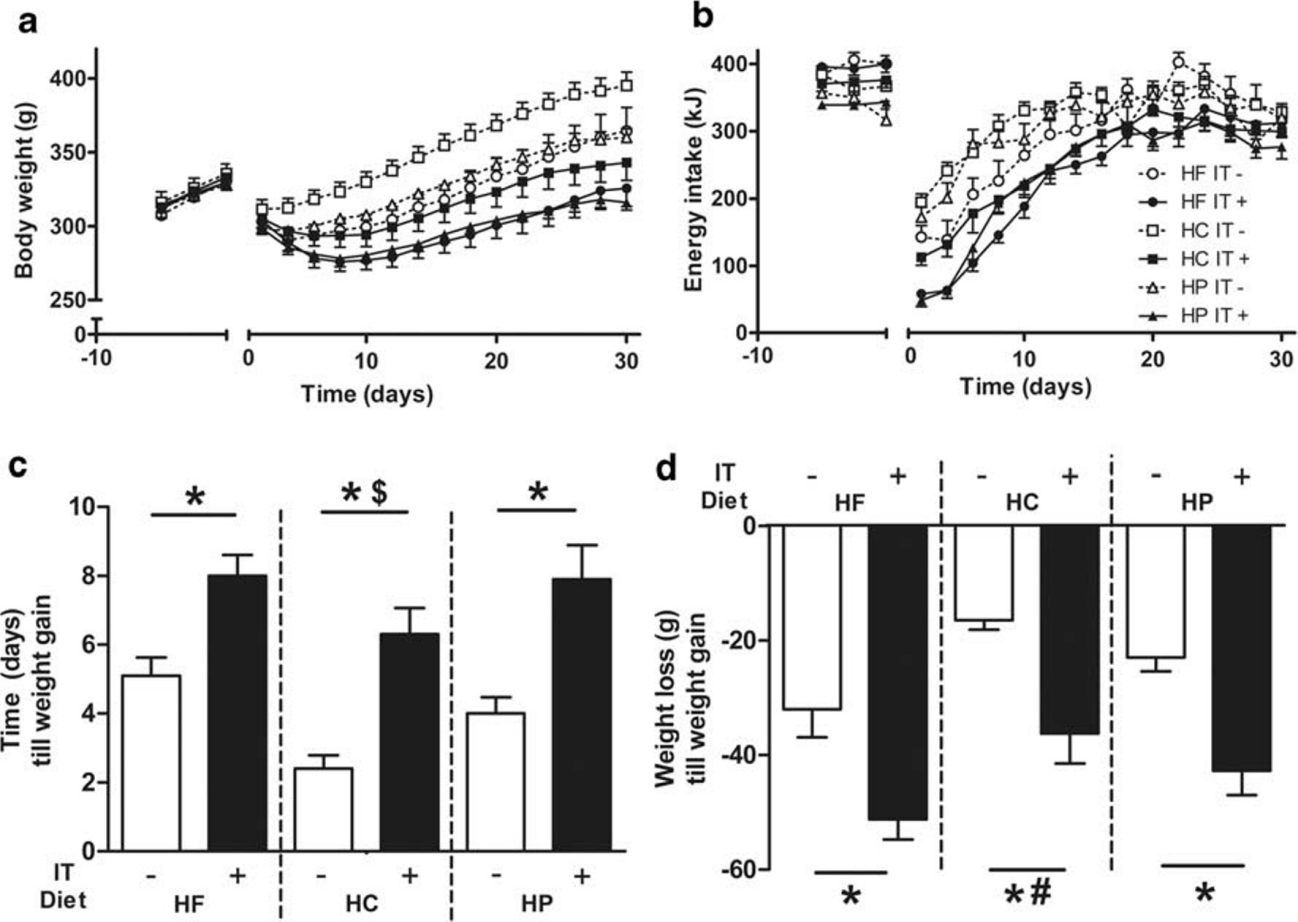
Effects of ileal transposition (IT+) and control surgery (IT−) in rats on a high-fat (HF; resp. *n* = 9, *n* = 8), high-carbohydrate (HC; resp. *n* = 9, *n* = 8), or a high-protein (HP; resp. *n* = 8, *n* = 9) diet on body weight (**a**), energy intake (**b**), post-surgery time (days) until rats started to gain weight again (**c**), and post-surgery total weight loss until rats started to gain weight again (**d**). For visibility, levels of body weight and energy intake in graphs **a** and **b** are averaged per 2 days. Levels of significance are only shown for figures **c** and **d**, with * indicating *p* < 0.01 for effects of ileal transposition (IT+) versus the control surgery (IT−), ^$^ indicating *p* < 0.01 for effect of diet relative to HF, and ^#^ indicating *p* < 0.05 for effect of diet relative to HF and HP.

Before surgery, 7-day weight gain differed between-diet groups (*F*_1,45_ = 9.74, *p* < 0.001), with HF feeding rats gaining most (33.4 ± 1.9 g) relative to HC feeding (30.8 ± 2.1 g) and HP feeding rats (22.6 ± 1.7 g). Following surgery, all groups lost weight immediately, with ileal transposition (IT+) rats losing more weight than sham-operated rats (IT−, *F*_1,45_ = 19.40, *p* = 0.004; i.e., assessed on the day of the lowest body weight following surgery). Then, rats started to gain weight again over the shown 30-day post-operative period (Fig. 1a), with IT+ rats gaining significantly less weight than IT− rats (*F*_1,45_ = 28.698, *p* < 0.001) on post-surgical day 30 relative to day 0. Additionally, diet affected weight gain after IT (*F*_2,45_ = 3.362, *p* < 0.05), with HC rats having higher body weights than HF and HP rats on post-surgical day 30.

Time until weight gain after surgery was significantly longer in IT+ than in IT− rats (*F*_1,45_ = 45.072, *p* < 0.0001, Fig. 1c). Effect of diet (*F*_2,45_ = 6.112, *p* < 0.01) was due to the shorter time until weight gain of HC rats (appr. 2 days) compared to HP and HF fed rats (appr. 4/5 days), although it only reached significance with HF rats (*p* < 0.01). Total weight loss after surgery until start of weight regain (Fig. 1d) was higher in IT+ than IT− rats (*F*_1,45_ = 37.391, *p* < 0.0001). Diet also affected weight loss (*F*_2,45_ = 7.568, *p* = 0.001), with HC feeding rats showing less weight loss than HP and HF feeding rats (*p* < 0.05) at the lowest level of body weight after surgery. Body weight at sacrifice (see Table 2) was lower in IT+ than IT− rats (*F*_1,45_ = 46.783, *p* < 0.0001). Diet also affected body weight (*F*_2,45_ = 9.747, *p* < 0.0001) with HP rats weighing less than HC rats (*p* < 0.01).

**Table 2:** Effects of ileal transposition (IT+) and control surgery (IT−) in rats on a high-fat (HF), high-carbohydrate (HC), or a high-protein (HP) diet on body weight and carcass constituents. Main effects of surgery and diet are indicated with *p* values or with non significance (ns). Lean body mass effects were not assessed with ANOVA, because of lack of normal distribution. IT effects were tested separately in diet groups. Between-diet group could not be assessed (na) for lean body mass

Diet	HF		HC		HP		Main effect	
Surgery	IT−	IT+	IT−	IT+	IT−	IT+	Surgery	Diet
Delta body weight (surgery-sacrifice) (g)	71.23 ± 13.55	13.43 ± 12.09	78.23 ± 8.59	29.21 ± 7.00	33.92 ± 5.84	-7.18 ± 9.04	IT+ < IT− *p* < 0.0001	HP < HC, *p* < 0.01
Body weight at sacrifice (g)	408.98 ± 18.06	354.80 ± 16.02	418.10 ± 13.13	367.1 ± 11.84	374.26 ± 6.82	326.45 ± 9.60	IT + < IT− *p* < 0.0001	HP < HC, *p* < 0.01
Total body fat (g)	69.25 ± 6.74	38.31 ± 5.94	58.12 ± 6.62	38.15 ± 3.87	44.76 ± 3.51	29.73 ± 3.14	IT+ < IT− *p* < 0.0001	HP < HF, *p* < 0.01
Visceral fat (g) (mesenteric + omental)	10.11 ± 1.18	5.48 ± 0.63	8.93 ± 0.91	6.44 ± 0.56	6.99 ± 0.42	4.55 ± 0.53	IT+ < IT− *p* < 0.0001	HP < HF, *p* < 0.05
Abdominal fat (g) (epidydimal, midline, and retroperitoneal)	23.99 ± 2.19	13.40 ± 2.24	20.61 ± 2.22	13.61 ± 1.47	15.50 ± 1.17	9.84 ± 1.20	IT+ < IT− *p* < 0.0001	HP < HF, *p* < 0.05
Total abdominal fat (g)	34.10 ± 3.30	18.88 ± 2.85	29.54 ± 3.08	20.05 ± 2.00	22.49 ± 1.50	14.40 ± 1.72	IT+ < IT− *p* < 0.0001	HP < HF/HC, *p* < 0.05
Subcutaneous fat (g)	26.51 ± 3.23	14.30 ± 2.75	20.29 ± 3.57	12.36 ± 1.64	15.01 ± 2.33	11.54 ± 1.60	IT+ < IT− *p* < 0.0001	HP < HF, *p* < 0.05
Inguinal fat (g)	8.64 ± 0.87	5.13 ± 0.69	8.30 ± 0.77	5.74 ± 0.61	7.26 ± 0.33	3.79 ± 0.35	IT+ < IT− *p* < 0.0001	ns
Lean body mass (g)	296.66 ± 10.63	292.10 ± 9.49	321.78 ± 13.16	302.34 ± 7.26	299.34 ± 4.39	278.28 ± 6.35	IT+ < IT− *p* < 0.05 only in HP gr.	na
Stomach	1.19 ± 0.05	1.24 ± 0.05	1.22 ± 0.04	1.21 ± 0.04	1.20 ± 0.02	1.22 ± 0.03	ns	ns
Upper duodenum segment (g)	0.42 ± 0.03	0.60 ± 0.05	0.32 ± 0.02	0.53 ± 0.04	0.39 ± 0.02	0.67 ± 0.06	IT+ > IT− *p* < 0.0001	HP > HC, *p* < 0.05
Lower duodenum segment (g)	0.22 ± 0.02	0.24 ± 0.03	0.15 ± 0.03	0.24 ± 0.04	0.19 ± 0.02	0.26 ± 0.05	IT+ > IT− *p* < 0.01	ns
Total duodenum (g)	0.64 ± 0.05	0.83 ± 0.07	0.46 ± 0.04	0.78 ± 0.04	0.58 ± 0.03	0.94 ± 0.08	IT+ > IT− *p* < 0.0001	ns
Transposed segment (g)	0.57 ± 0.12	2.10 ± 0.29	0.77 ± 0.26	1.80 ± 0.18	0.50 ± 0.06	1.60 ± 0.10	IT + > IT− *p* < 0.0001	ns
Jejunoileum (g)	2.37 ± 0.18	3.75 ± 0.31	2.32 ± 0.24	3.34 ± 0.19	2.08 ± 0.10	3.66 ± 0.17	IT+ > IT− *p* < 0.0001	ns
Distal ileum segment (g)	0.36 ± 0.05	0.64 ± 0.06	0.42 ± 0.03	0.53 ± 0.05	0.40 ± 0.03	0.63 ± 0.04	IT+ > IT− *p* < 0.0001	ns
Total small intestine (g)	3.95 ± 0.41	7.32 ± 0.73	3.97 ± 0.58	6.75 ± 0.41	3.55 ± 0.23	6.82 ± 0.01	IT+ > IT−r *p* < 0.0001	ns
Cecum and colon with content (g)	3.95 ± 0.13	6.02 ± 0.50	5.23 ± 0.52	6.08 ± 0.34	4.70 ± 0.22	6.14 ± 0.41	IT+ > IT− *p* < 0.01	ns
Pancreas (g)	1.24 ± 0.03	1.42 ± 0.09	1.24 ± 0.07	1.47 ± 0.04	1.36 ± 0.03	1.48 ± 0.07	IT+ > IT− *p* < 0.0001	ns
Kidneys (g)	2.61 ± 0.13	2.42 ± 0.10	2.62 ± 0.11	2.51 ± 0.09	2.80 ± 0.09	2.48 ± 0.07	IT+ < IT−*p* < 0.01	ns
Liver (g)	12.61 ± 0.69	13.22 ± 0.68	14.37 ± 0.64	14.52 ± 0.67	13.41 ± 0.61	12.39 ± 0.64	ns	ns

### Energy Intake

Average daily energy intake (EI, Fig. 1b) before surgery was affected by diet (*F*_2,45_ = 46.3, *p* < 0.0001), with highest EI in the HF rats 395.28 ± 5.39 kJ per day, followed by HC rats (375.77 ± 6.45 kJ, HF vs. HC *p* < 0.001) and HP rats (341.87 ± 4.32 kJ, HP vs. HC and HF, *p* < 0.001). Overall, IT+ rats had decreased EI compared to IT− rats (*F*_1,45_ = 45.8, *p* < 0.0001) during 30 days post-operatively. During this period, diet also affected EI (*F*_2,45_ = 3.362, *p* < 0.05); however, post hoc analysis showed no significance between the diet groups.

Dividing the post-operative 30-day interval into 10-day periods revealed that cumulative EI was lower in IT+ rats compared to IT− rats in the first (*F*_1,45_ = 63.309, *p* < 0.0001), second (*F*_1,45_ = 23.535, *p* < 0.0001), and third (*F*_1,45_ = 9.294, *p* < 0.01) 10-day periods. Diet also affected EI in the first (*F*_2,45_ = 7.813, *p* < 0.001) and second (*F*_2,45_ = 3.666, *p* < 0.05) 10-day periods (with HC rats ingesting the most and HF rats ingesting the least), but not anymore during the third 10-day period. During the period of blood sampling (between days 40 and 50), IT+ rats had lower EI than IT− rats (*F*_1,42_ = 4.601, *p* < 0.05), but again no effect of diet was observed (data not shown).

### Energy Efficiency

**Fig. 2:**
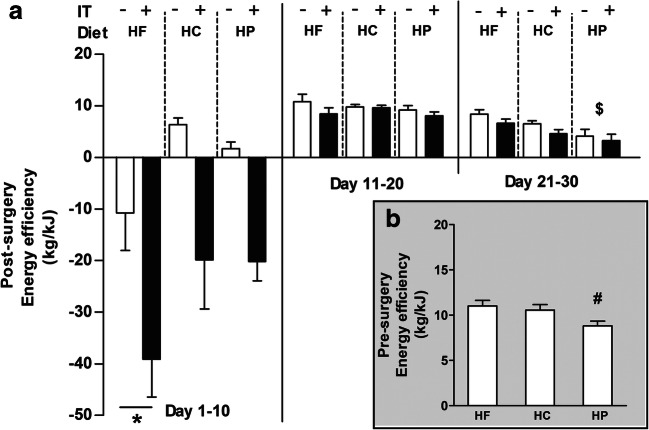
Effects of ileal transposition (IT+) and control surgery (IT−) in rats on a high-fat (HF; resp. *n* = 9, *n* = 8), high-carbohydrate (HC; resp. *n* = 9, *n* = 8) or a high-protein (HP; resp. *n* = 8, *n* = 9) diet on energy efficiency in 10-day periods (panel **a**). The insert (**b**) shows baseline energy efficiency in rats on the three different diets. * denotes significant difference relative to HC and HP (*p* < 0.05), ^$^ denotes significant difference (*p* < 0.001) relative to HF. ^#^ denotes significant different (*p* < 0.05) relative to HC and HF

Before surgery, diet affected energy efficiency (*F*_2,45_ = 5.240, *p* < 0.05), with significantly lower values of HP rats compared to HF (*p* < 0.01) and HC rats (*p* < 0.05; see insert b in Fig. 2). After surgery, IT+ rats showed a large decrease in energy efficiency (Fig. 2a) during the first 10-day period post-operative compared to the IT− groups (*F*_1,45_ = 23.703, *p* < 0.0001). The effect of diet (*F*_2,45_ = 5.364 *p* < 0.008) was mainly due to lower energy efficiency levels of HF relative to HC and HP rats (*p* < 0.05). During the second and third 10-day periods, IT+ showed similar energy efficiency as IT− in all different diet groups. During the third 10-day period diet affected energy efficiency (*F*_2,45_ = 7.590, *p* < 0.001) with HP rats having significantly lower energy efficiency than HF rats (*p* < 0.001).

### Gastrointestinal Hormone Levels

During baseline conditions (i.e., before meal exposure), IT+ rats had decreased plasma levels of insulin (*F*_1,44_ = 5.737, *p* = 0.021, panel g, with post hoc significance in HF feeding rats), and increased plasma levels of neurotensin (NT; *F*_1,44_ = 4.651, *p* = 0.037, panel j) and PYY (*F*_1,44_ = 14.650, *p* = < 0.0001, panel m, with post hoc significance in HF and HC feeding rats).

Surgery and diet affected meal-induced excursions (AUC_0–60_) of plasma GIP (surgery *F*_1,44_ = 12.311, *p* = 0.001; diet *F*_2,44_ = 7.473, *p* = 0.002; panel b), plasma GLP-1 (surgery *F*_1,44_ = 23.632, *p* < 0.0001; diet *F*_2,44_ = 3.409, *p* = 0.042; panel e), and plasma insulin (surgery *F*_1,44_ = 10.832, *p* = 0.002; diet *F*_2,44_ = 3.557, *p* = 0.037; panel h). AUC_0–60_ of plasma neurotensin (*F*_1,44_ = 33.187, *p* < 0.0001, panel k) and PYY (*F*_1,44_ = 73.326, *p* < 0.0001, panel n) were only affected by surgery. Post hoc analysis of AUC_0–60_ showed that IT+ decreased GIP (only in the HF group) and insulin (only in the HC group), and increased GLP-1, neurotensin, and PYY (IT+ vs. IT− *p* < 0.05–*p* < 0.001). Between-diet analysis showed overall decreased AUC_0–60_ responses of GIP in the HP diet group relative to both HC and HF diet groups. Decreased AUC_0–60_ of GLP-1 and increased insulin AUC_0–60_ was seen when comparing the HP diet group with the HC diet group.

**Fig. 3:**
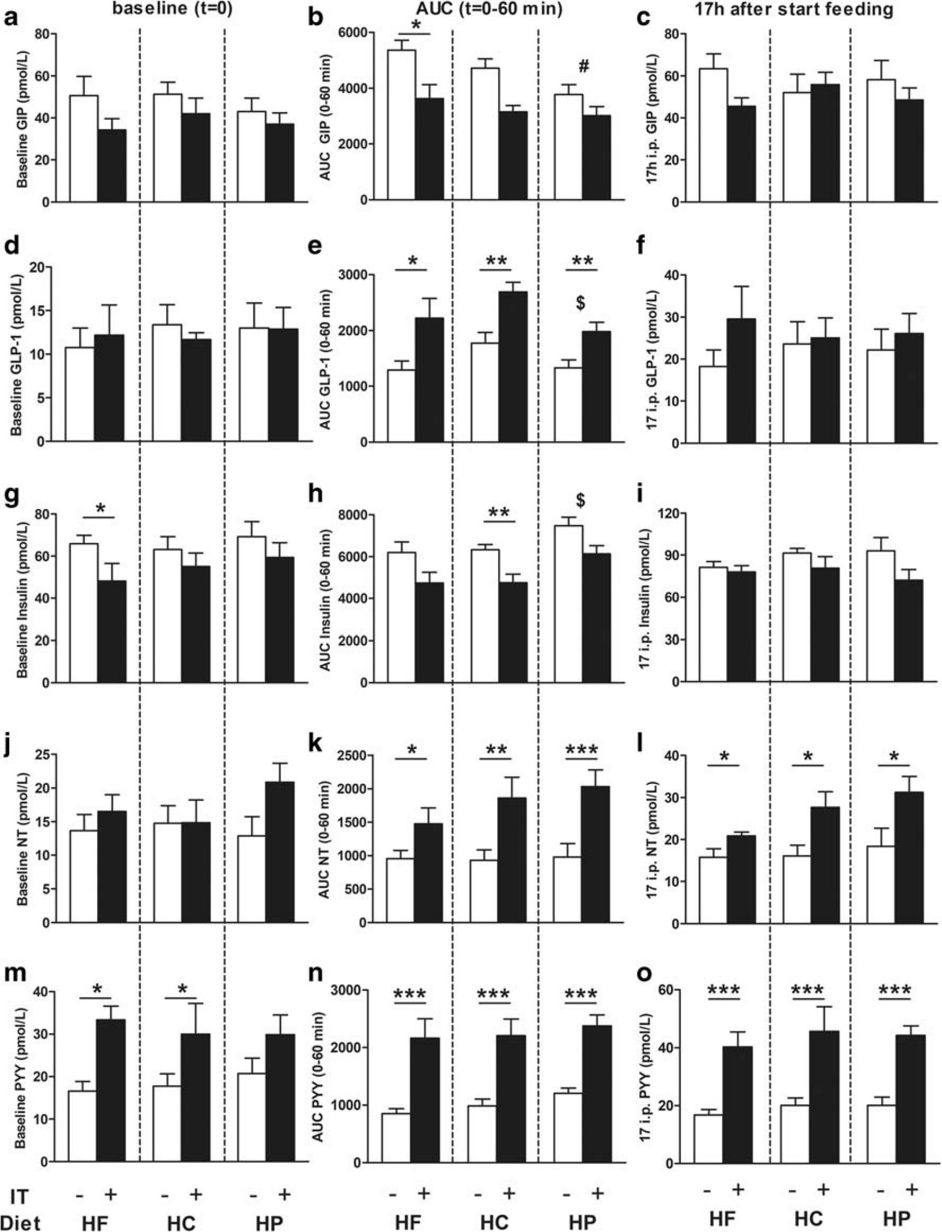
Effects of ileal transposition (IT+) and control surgery (IT−) in rats feeding a high-fat (HF; resp. *n* = 9, *n* = 8), a high-carbohydrate (HC; resp. *n* = 8, *n* = 8) or a high-protein (HP; resp. *n* = 8, *n* = 9) diet on baseline hormone levels (panels **a**, **d**, **g**, **j**, **m**), incremental area under the curve responses over the course of 60 min (AUC_0–60_) during and following a meal (panels **b**, **e**, **h**, **k**, **n**), and 17-h post-ingestive levels (panels **c**, **f**, **i**, **l**, **o**). Level of significance of IT surgery within diet groups are depicted by * (*p* < 0.05), ** (*p* < 0.01), or *** (*p* < 0.001). Effects of diet between HP and HC groups are indicated by ^$^ (*p* < 0.05). Effects of diet between HP and HC and HP and HF are indicated by ^#^ (*p* < 0.05)

**Fig. 4:**
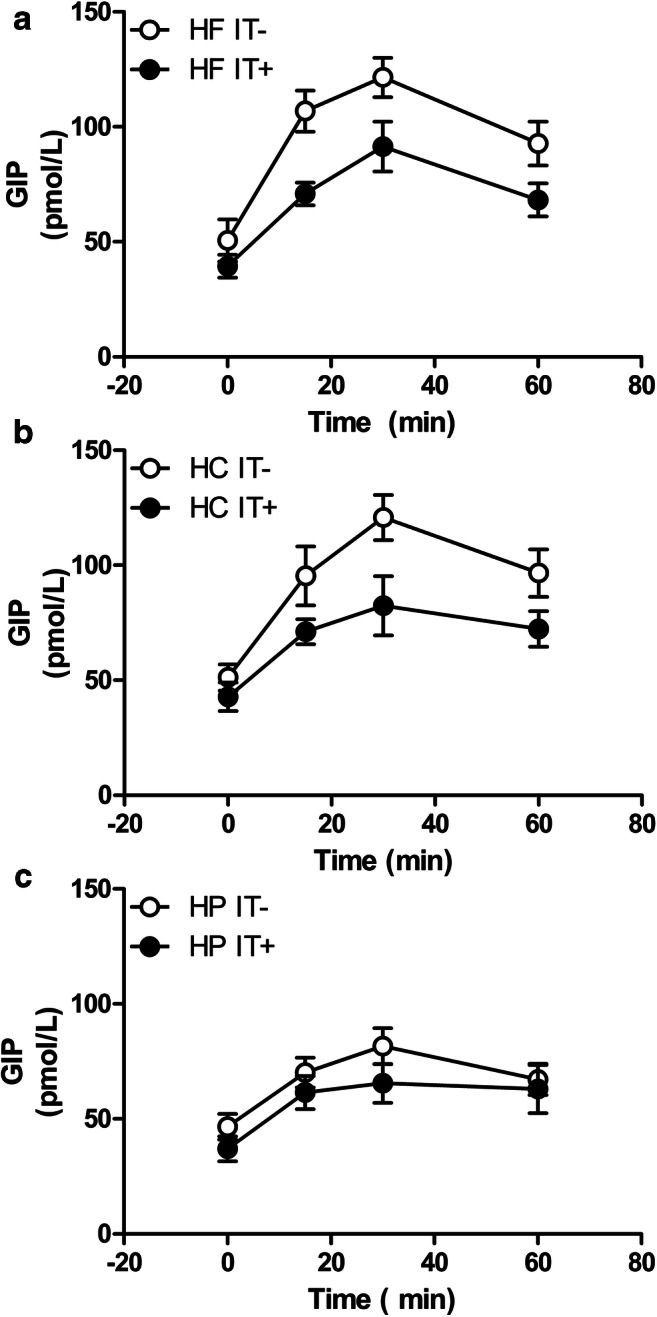
Effects of ileal transposition (IT+) and control surgery (IT−) in rats feeding a high-fat (HF; resp. *n* = 9, *n* = 8), a high-carbohydrate (HC; resp. *n* = 8, *n* = 8) or a high-protein (HP; resp. *n* = 8, *n* = 9) diet on plasma GIP levels before (0) and during/after (15 min, 30 min, 45 min, 60 min) a meal

**Fig. 5:**
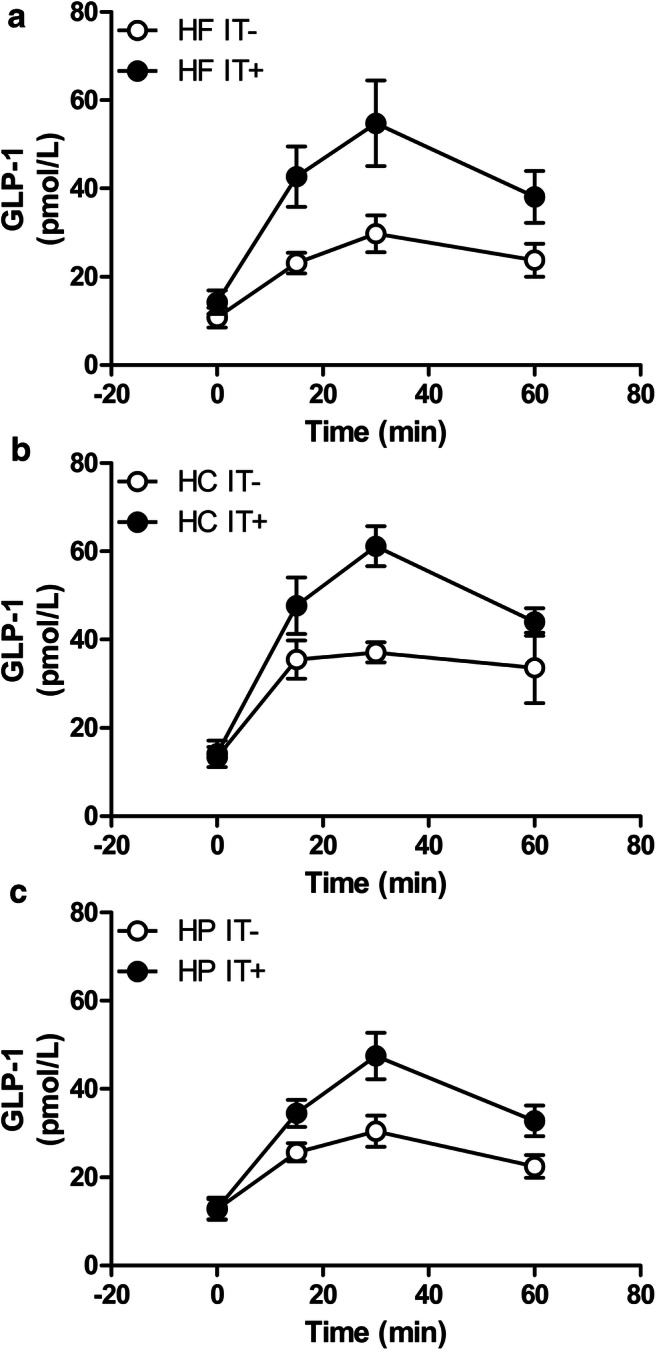
Effects of ileal transposition (IT+) and control surgery (IT−) in rats feeding a high-fat (HF; resp. *n* = 9, *n* = 8), a high-carbohydrate (HC; resp. *n* = 8, *n* = 8) or a high-protein (HP; resp. *n* = 8, *n* = 9) diet on plasma GLP-1 levels before (0) and during/after (15 min, 30 min, 45 min, 60 min) a meal

**Fig. 6:**
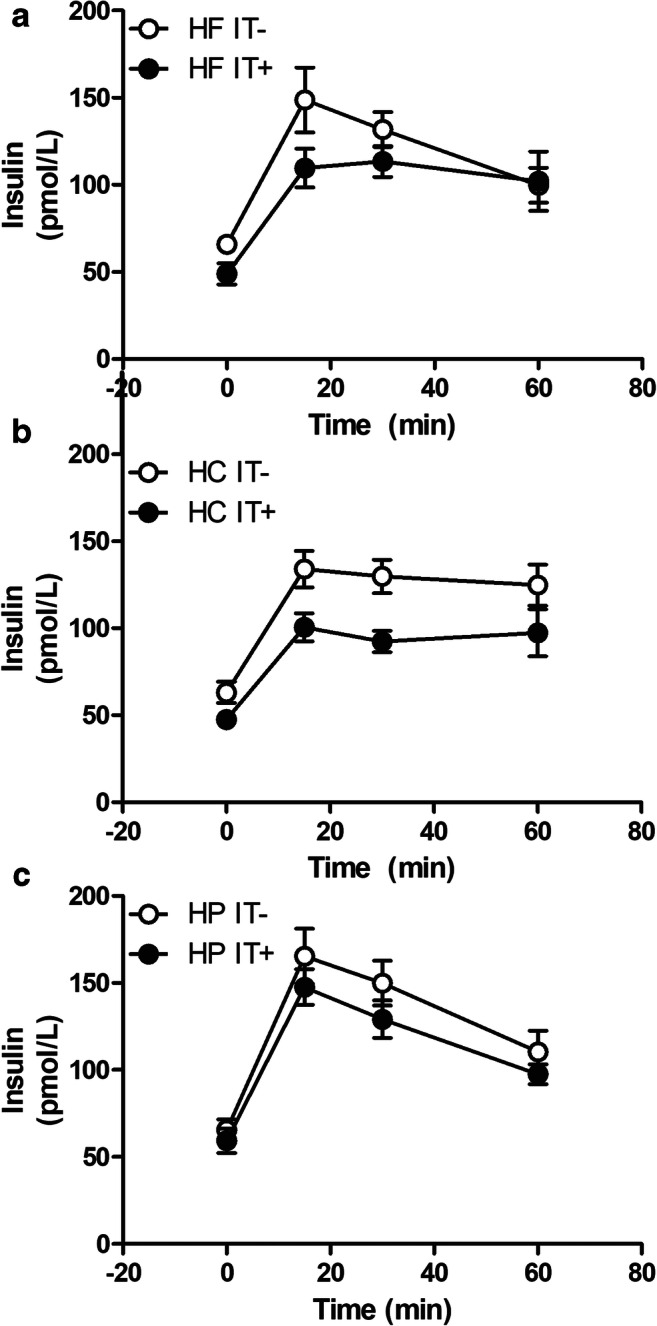
Effects of ileal transposition (IT+) and control surgery (IT−) in rats feeding a high-fat (HF; resp. *n* = 9, *n* = 8), a high-carbohydrate (HC; resp. *n* = 8, *n* = 8) or a high-protein (HP; resp. *n* = 8, *n* = 9) diet on plasma insulin levels before (0) and during/after (15 min, 30 min, 45 min, 60 min) a meal

**Fig. 7:**
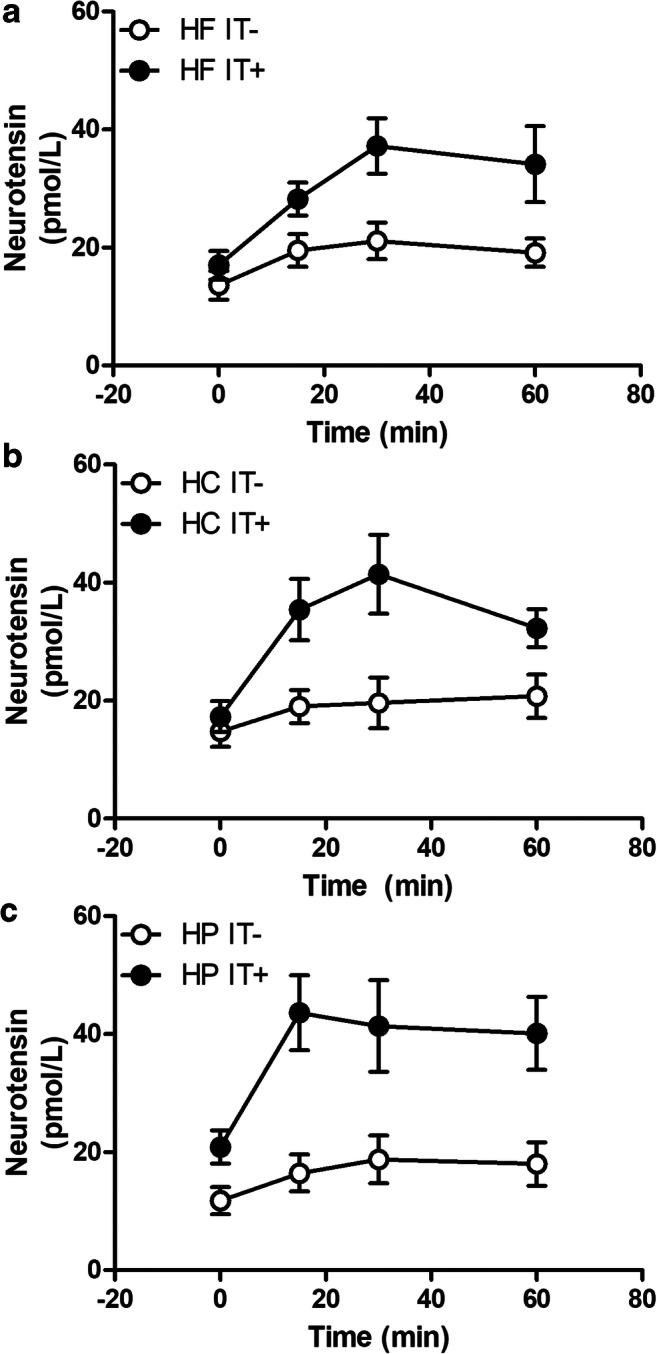
Effects of ileal transposition (IT+) and control surgery (IT−) in rats feeding a high-fat (HF; resp. *n* = 9, *n* = 8), a high-carbohydrate (HC; resp. *n* = 8, *n* = 8) or a high-protein (HP; resp. *n* = 8, *n* = 9) diet on plasma neurotensin levels before (0) and during/after (15 min, 30 min, 45 min, 60 min) a meal

**Fig. 8:**
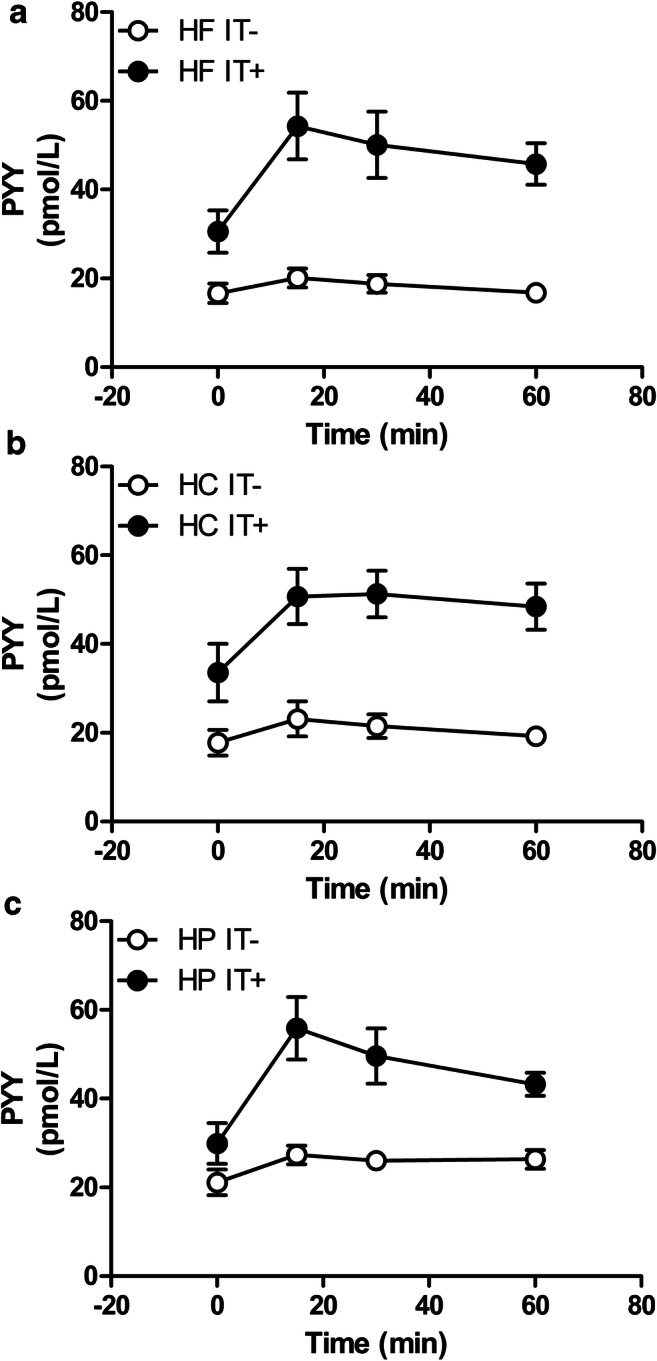
Effects of ileal transposition (IT+) and control surgery (IT−) in rats feeding a high-fat (HF; resp. *n* = 9, *n* = 8), a high-carbohydrate (HC; resp. *n* = 8, *n* = 8) or a high-protein (HP; resp. *n* = 8, *n* = 9) diet on plasma PYY levels before (0) and during/after (15 min, 30 min, 45 min, 60 min) a meal

Seventeen-hour post-ingestive plasma hormone levels showed an overall surgery effect, with IT+ rats showing elevated plasma GLP-1 (*F*_1,44_ = 4.603, *p* = 0.037, panel f), plasma neurotensin (*F*_1,44_ = 20.428, *p* < 0.0001, panel l) and plasma PYY (*F*_1,44_ = 60.288, *p* < 0.0001, panel o), and reduced plasma insulin (*F*_1,44_ = 5.470, *p* = 0.024, panel i). Post hoc analysis revealed increases in IT+ rats of plasma neurotensin and PYY in all diet groups; however, this was not the case for GLP-1 and insulin. No effect was found on 17-h post-ingestive levels of GIP (Fig. 3).

### Body Composition (Table 2)

IT+ resulted in lower adipose tissue pads in all assessed body regions (mesenteric fat content *F*_1,45_ = 34.097, *p* < 0.0001, total abdominal fat content *F*_1,45_ = 35.459, *p* < 0.0001, subcutaneous fat content *F*_1,45_ = 17.323, *p* < 0.0001, inguinal fat content *F*_1,45_ = 46.155, *p* < 0.0001; Table 2), and an overall decreased body fat content (*F*_1,45_ = 33.670, *p* < 0.0001) relative to IT− rats. Lean body mass (LBM) was not normally distributed across all groups, and we therefore analyzed the data separately per diet group (in which the data was normally distributed). IT+ rats had lower LBM relative to IT− rats only in the rats feeding a HP diet (*p* < 0.003). Post hoc analysis showed a difference between IT+ and IT− rats feeding the HP and HF diet on all different adipose tissue contents (*p* < 0.05–*p* < 0.01), except for the inguinal region in the HP feeding rats. HP showed only a significant difference with the HC diet group in subcutaneous adipose fat content (*p* < 0.05). Diet affected total body fat content (*F*_2,45_ = 4.619, *p* = 0.015), with highest levels in HF feeding rats and lowest in HP feeding rats (HF vs HP, *p* = 0.031).

All collected gastrointestinal tract parts showed increased wet weights after IT+ compared to IT−, with the exception of the stomach. Starting anatomically from the top, the upper duodenum and lower duodenum showed increased wet weights, resulting in higher wet weight of the total duodenum (*F*_1,45_ = 40.982, *p* < 0.0001). The transposed segment, the jejunoileum, and lowest segment of the ileum showed higher wet weights (*F*_1,45_ = 75.864, *p* < 0.0001; *F*_1,45_ = 32.944, *p* < 0.0001 respectively), resulting in overall higher wet weight of the small intestine (*F*_1,45_ = 94.666, *p* < 0.0001). Finally, IT+ resulted in increased wet weights of the cecum and colon (*F*_1,45_ = 27.423, *p* < 0.0001). Between-diet analysis revealed that the HP group showed higher wet weight of the upper part of the duodenum compared to the HF group (*p* < 0.05).

IT+ resulted in heavier pancreas weight (*F*_1,45_ = 15.667, *p* < 0.0001), lighter kidney weight (*F*_1,45_ = 8.522, *p* < 0.01) and no differences in liver weight, when compared to the IT− group. Diet had an effect on liver weight (*F*_2,45_ = 4.631, *p* < 0.05). Detailed post hoc analysis of the adipose tissue content, wet weight of the gastrointestinal tract, and organs in the abdominal cavity are shown in Table 2.

### Regression Analysis

In the stepwise linear regression analysis, cumulative energy intake during the post-operative 30-day period was highly positively correlated to the observed body weight change after surgery and predicted its variation by 82.3% (*B* = 2.743, *R*^2^ = 0.823, *p* < 0.0001). This model was improved significantly by diet to finally explain 86.0% of the variation in weight change following surgery.

We subsequently investigated whether gastrointestinal hormonal factors could be explained, in addition to the diet and surgery effects, by the variation (1) in energy intake over the course of the 30-day post-operative (recovery) period after surgery, or (2) the energy intake during the time of blood sampling. In most of the models, surgery type was the main factor explaining variation in the gut hormone levels at baseline, the AUC during/after a meal, and 17-h post-ingestive sampling point after start of feeding, without any significant contributions of energy intake. Only in the case of the post-ingestive 17-h PYY levels, the cumulative energy intake in the post-operative 30-day period significantly contributed to the factor “surgery” together explaining 61.3% of the variation in 17-h PYY levels (*B*_surgery_ = 18.209, *B*_energy intake_ = − 0.674, *R*^2^ = 0.613, *p* < 0.001). Removal of surgery from the independence list still caused energy intake during the post-operative 30-day period to significantly explain 47.4% of the variation in 17-h PYY levels (*B*_energy intake_ = − 1.392. *R*^2^ = 0.474, *p* < 0.001) in a negative direction (i.e., the higher the 17-h PYY levels, the lower the intake).

## Discussion

In the present study, we confirmed that ileal transposition (IT+) in rats causes reduced energy intake [2], augmented weight loss [8–10], and altered gut hormone responses to nutrients [11, 15, 32]. The novel finding is that IT+ rats on the HP diet had lowest intake and highest surgery-induced weight loss among the different diets, and those on the HC diet had the highest intake and lowest weight loss. Apart from a transiently reduced energy efficiency during the first 10 days following IT+ surgery (which was most profound in the HF rats) compared to IT− surgery, energy efficiency became normalized again in IT+ rats, and non-distinguishable from IT− rats. This suggests that the primary mechanism for weight loss and reduced weight maintenance is energy intake driven. The fact that energy intake was the main driving factor for differences in body weight between IT+ and IT− rats was also shown by the regression analysis, in which 82.3% of the weight change was explained by energy intake alone, without further contribution of the factor “surgery” to this regression model.

Immediately following IT+ as well as IT− surgery, energy intake and energy efficiency were lowest on the HF diet. This was somewhat of a surprise, since the HF diet was ingested the most of all diets before surgery, and that is consistent with other studies [33, 34]. Dietary fat causes low-grade gastrointestinal inflammation in rats [35, 36] and in humans [37, 38], which is diffused by a layer of visceral fat [39]. It may be speculated that loss of visceral fat shortly after surgery may compromise the endotoxemia barrier, which could then underlie higher sickness behavior after surgery in the HF feeding rats [40]. In the case of the HC and HP diets, where fat content was 25% less than in the HF diet, such a problem may have occurred less. Towards the later stage of the study, however, HF feeding rats caught up and surpassed body weight of HP feeding rats, and in fact had the highest fat deposition at every fat pad studied on the day of sacrifice.

The transposed ileal segments of IT+ rats obviously contained a large number of PYY, GLP-1, and oxyntomodulin secreting endocrine L cells, witnessed by higher levels of these anorexigenic gut hormones. The enterotropic effects seen in several gut segments in response to IT+ most likely results from increased secretion of glucagon-like peptide 2 (GLP-2). Although we did not measure this hormone, its levels would be expected to be increased as it is co-produced with GLP-1 from proglucagon and is the only enteric hormone known to have marked growth effects on the small bowel mucosa [41]. Additionally, we noticed that IT+ rats also had heavier pancreas weights, which was probably due to an increase in exocrine parenchyma caused by higher levels of CCK [42]. Although we did not assess CCK profiles, levels of CCK appear to be upregulated by bariatric surgeries [43].

Apart from the finding that meal-induced levels of PYY, GLP-1 and Neurotensin were elevated by IT, we did not observe major differences between-diet groups in these gut hormone responses. The relative dietary increases of macronutrients in the present study (50% versus 25%) were apparently too small to find (meal-induced) differences in gut hormone responses comparable to those that were reported in other studies using macronutrient-enriched diets [15, 26, 44–47]. Additional regression analysis showed that “surgery-type” (but not “diet-type”) and 30-day post-operative cumulative energy intake highly explained variation in 17-h post-ingestive PYY levels. When “surgery-type” was taken out of the model, the 17-h post-ingestive PYY level was negatively correlated to 30-day cumulative energy intake and explaining 47.4% of its variation. While our data seem to be in line with the fact that PYY is implicated in the long-term regulation of body weight and energy intake in rodents [48, 49] and humans [50], such a relation between food intake and PYY levels was not observed in the period during the actual blood sampling phase (between day 40 and 50, when rats were weight stable). Because PYY is an anorexigenic gut hormone, one explanation is that the 17-h post-ingestive PYY level during weight stability could be regarded as a unique signature of rats that -on top of the effect of IT surgery- was already present in the phase right after surgery. While this hypothesis should be tested in future studies, it may be of additional interest to investigate whether or not post-ingestive PYY levels could serve as a marker of weight change directly after surgery. A more general role for PYY in weight regain is consistent with the fact that high levels of PYY predict lower weight and appetite regain in patients that recover from weight loss associated with tuberculosis [51]. Although the PYY3–36 amide form is the predominant circulating form [52] as well as the only form that reliably reduces energy intake [53], one point of caution here is the fact that the assay used in this study did not discriminate between PYY1–36 and PYY3–36 amidated forms.

During the pre-surgical period the HP diet was eaten in the least amount, causing lowest weight gain among diet groups. Like fats, proteins have also been hypothesized to activate the ileal brake [23, 54], but high-protein diets may be more satiating than HF diets or HC diets [17, 20, 55, 56]. Diets in the current study contained casein, a dairy protein, which has been shown to reduce energy intake in rats [57, 58] due to increased satiety [17, 57, 59] rather than low palatability [58, 60] or taste aversion [61]. After IT surgery, however, the extent of weight loss and length of the weight loss period of the HP rats were less than those of HF rats. A lowest level of energy efficiency was nevertheless found in HP feeding rats towards the end of the post-surgery (recovery) phase (days 20–30), indicating that protein exerted a long-term stimulatory effect on metabolism. Perhaps as a result of this, IT+ rats feeding a HP diet had the least amount of body fat at the end of the study, and this was associated with a reduction in lean mass. At present, we do not have an explanation for this effect, but it seems consistent with other bariatric data [62]. If any, we may rule out alteration in gut hormone responses contributing to these protein-mediated reductions in body fat and lean mass following IT surgery.

A point that deserves attention is the fact that we offered the diets liquified. Rats and humans consume more of a liquid diet than that of a solid one [63, 64]. After surgery (both after IT+ and IT-) HC rats ate the most, suggesting that a weaker activation of ileal brake or faster recovery from surgery with a diet containing more carbohydrates. The energy efficiency values are also in line with faster recovery, since HC control rats had positive energy efficiencies even immediately after surgery, resulting in a shorter recovery period with less weight loss. Even though HC transposed animals had negative energy efficiency in the first 10 days, they recovered faster and lost less weight than HF and even HP transposed animals. The carbohydrate fraction of the diet consisted of 40% sucrose, thus probably giving the animals a very appealing taste. Rats have been shown to consume significantly more calories (compared to standard diet) if offered a high-sucrose or high-fat/high-sugar (cafeteria) diet [65–67]. Both in rats [68] and in humans, sucrose sweetened beverages have been shown to be more obesogenic than other types of beverages [69] and liquid diet as solid food replacement [70, 71] blunt the natural post-prandial decline of hunger, increase subsequent food intake, and potentially represent a risk for exaggerated positive energy balance in humans and in rats [72].

In summary, we showed that IT caused transient body weight loss, which was the least in HC feeding rats, and highest in HF and HP feeding rats. Energy efficiency dropped immediately after surgery but normalized after 10 days post-surgery, not contributing significantly to differences in weight change between IT+ and IT− rats. Regression analysis showed that energy intake alone predicted body weight changes by more than 80%, which was certainly influenced by surgery type and to a lesser degree by diet. Post-ingestive PYY levels explained reduced post-surgery weight change by 47% irrespective of IT. Our data imply that ingesting a HP diet is most effective to support body weight loss and avoid body fat regain after ileal transposition. The downside of the latter effect is that it appears to be associated with a loss of lean body mass.

## References

[1] Dixon JB, le Roux CW, Rubino F, Zimmet P (2012). Bariatric surgery for type 2 diabetes. Lancet..

[2] Ndumele CE, Matsushita K, Lazo M, Bello N, Blumenthal RS, Gerstenblith G (2016). Obesity and subtypes of incident cardiovascular disease. J Am Heart Assoc.

[3] Grundy SM (2016). Metabolic syndrome update. Trends Cardiovasc Med.

[4] Azvolinsky A. The obesity–cancer link: a growing connection. J Natl Cancer Inst. 2016;108(10):djw243. 10.1093/jnci/djw243 27737916

[5] Finkelstein EA, Trogdon JG, Cohen JW, Dietz W (2009). Annual medical spending attributable to obesity: payer-and service-specific estimates. Health Aff (Millwood).

[6] Weiss EC, Galuska DA, Kettel Khan L, Gillespie C, Serdula MK (2007). Weight regain in U.S. adults who experienced substantial weight loss,1999-2002. Am J Prev Med.

[7] Kraschnewski JL, Boan J, Esposito J, Sherwood NE, Lehman EB, Kephart DK, Sciamanna CN (2010). Long-term weight loss maintenance in the United States. Int J Obes.

[8] Fichtner C, Koopmans HS, Sclafani A, Aravich PF (1982). The effects of ileal transposition on food intake and bodyweight loss in VMH-obese rats. Am J Clin Nutr.

[9] Ramzy AR, Nausheen S, Chelikani PK (2014). Ileal transposition surgery produces ileal length-dependent changes in food intake, bodyweight, gut hormones and glucose metabolism in rats. Int J Obes.

[10] Koopmans HS, Ferri GL, Sarson DL, Polak JM, Bloom SR (1982). The effects of ileal transposition and jejunoileal bypass on food intake and GI hormone levels in rats. Physiol Behav.

[11] Chelikani PK, Shah IH, Taqi E, Sigalet DL, Koopmans HH (2010). Comparison of the effects of Roux-en-Y gastric bypass and ileal transposition surgeries on food intake, bodyweight, and circulating peptide YY concentrations in rats. Obes Surg.

[12] Chen DC, Stern JS, Atkinson RL (1990). Effects of ileal transposition on food intake, dietary preference, and weight gain in Zucker obese rats. Am J Physiol Regul Integr Comp Physiol.

[13] Read NW, McFarlane A, Kinsman RI, Bates TE, Blackhall NW, Farrar GBJ, Hall JC, Moss G, Morris AP, O'Neill B (1984). Effect of infusion of nutrient solutions into the ileum on gastrointestinal transit and plasma levels of neurotensin and enteroglucagon. Gastroenterology..

[14] Spiller RC, Trotman IF, Higgins BE, Ghatei MA, Grimble GK, Lee YC, Bloom SR, Misiewicz JJ, Silk DB (1984). The ileal brake--inhibition of jejunal motility after ileal fat perfusion in man. Gut..

[15] Batterham RL, Heffron H, Kapoor S, Chivers JE, Chandarana K, Herzog H, le Roux CW, Thomas EL, Bell JD, Withers DJ (2006). Critical role for peptide YY in protein-mediated satiation and body-weight regulation. Cell Metab.

[16] Martens EA, Tan SY, Dunlop MV, Mattes RD, Westerterp-Plantenga MS (2014). Protein leverage effects of beef protein on energy intake in humans. Am J Clin Nutr.

[17] Bensaïd A, Tomé D, L'Heureux-Bourdon D, Even P, Gietzen D, Morens C, Gaudichon C, Larue-Achagiotis C, Fromentin G (2003). A high-protein diet enhances satiety without conditioned taste aversion in the rat. Physiol Behav.

[18] Zapata RC, Singh A, Chelikani PK (2018). Peptide YY mediates the satiety effects of diets enriched with whey protein fractions in male rats. FASEB J.

[19] Leidy HJ, Clifton PM, Astrup A, Wycherley TP, Westerterp-Plantenga MS, Luscombe-Marsh ND, Woods SC, Mattes RD (2015). The role of protein in weight loss and maintenance. Am J Clin Nutr.

[20] Feinman RD, Pogozelski WK, Astrup A, Bernstein RK, Fine EJ, Westman EC, Accurso A, Frassetto L, Gower BA, McFarlane S, Nielsen JV, Krarup T, Saslow L, Roth KS, Vernon MC, Volek JS, Wilshire GB, Dahlqvist A, Sundberg R, Childers A, Morrison K, Manninen AH, Dashti HM, Wood RJ, Wortman J, Worm N (2015). Dietary carbohydrate restriction as the first approach in diabetes management: critical review and evidence base. Nutrition..

[21] Westerterp-Plantenga MS, Lemmens SG, Westerterp KR (2012). Dietary protein – its role in satiety, energetics, weight loss and health. Br J Nutr.

[22] Lin HC, Wang L, Zhao X-T, McCamish MA, Miller RH (1997). Intestinal transit and absorption of soy protein in dogs depend on load and degree of protein hydrolysis. J Nutr.

[23] Maljaars PW, Peters HP, Mela DJ, Masclee AA (2008). Ileal brake: a sensible food target for appetite control. A review. Physiol Behav.

[24] Van Citters GW, Lin HC (1999). The ileal brake: a fifteen-year progress report. Cur Gastroenterol Rep.

[25] Van Citters GW, Lin HC (2000). Ileal brake: neuropeptidergic control of intestinal transit. Curr Gastroenterol Rep.

[26] Adrian TE, Ferri GL, Bacarese-Hamilton AJ, Fuessl HS, Polak JM, Bloom SR (1985). Human distribution and release of a putative new gut hormone, peptide YY. Gastroenterology..

[27] Uttenthel LO, Ghiglione M, George SK, Bishop AE, Polak JM, Bloom SR (1985). Molecular forms of glucagon-like peptide-1 in human pancreas and glucagonomas. J Clin Endocrinol Metab.

[28] Sarson DL, Bryant MG, Bloom SR (1980). A radioimmunoassay of gastric inhibitory polypeptide in human plasma. J Endocrinol.

[29] Blackburn AM, Bloom SR (1979). A radioimmunoassay for neurotensin in human plasma. J Endocrinol.

[30] Adrian TE, Gariballa S, Parekh KA, Thomas SA, Saadi H, Al Kaabi J (2012). Rectal taurocholate increases L cell and insulin secretion, and decreases blood glucose and food intake in obese type 2 diabetic volunteers. Diabetologia..

[31] Björntorp P, Yang MU (1982). Refeeding after fasting in the rat: effects on body composition and food efficiency. Am J Clin Nutr.

[32] Gaitonde S, Kohli R, Seeley R (2012). The role of the gut hormone GLP-1 in the metabolic improvements caused by ileal transposition. J Surg Res.

[33] Sharma S, Fernandes MF, Fulton S (2013). Adaptations in brain reward circuitry underlie palatable food cravings and anxiety induced by high-fat diet withdrawal. Int J Obes.

[34] Pickering C, Alsiö J, Hulting A-L, Schiöth HB (2009). Withdrawal from free-choice high-fat high-sugar diet induces craving only in obesity-prone animals. Psychopharmacology..

[35] Denver P, Gault VA, McClean PL (2018). Sustained high-fat diet modulates inflammation, insulin signalling and cognition in mice and a modified xenin peptide ameliorates neuropathology in a chronic high-fat model. Diabetes Obes Metab.

[36] Gil-Cardoso K, Ginés I, Pinent M, Ardévol A, Terra X, Blay M (2017). A cafeteria diet triggers intestinal inflammation and oxidative stress in obese rats. Br J Nutr.

[37] Pendyala S, Walker JM, Holt PR (2012). A high-fat diet is associated with endotoxemia that originates from the gut. Gastroenterology.

[38] Ruiz-Núñez B, Pruimboom L, Dijck-Brouwer DA, Muskiet FA (2013). Lifestyle and nutritional imbalances associated with Western diseases: causes and consequences of chronic systemic low-grade inflammation in an evolutionary context. J Nutr Biochem.

[39] Wernstedt Asterholm I, Tao C, Morley TS, Wang QA, Delgado-Lopez F, Wang ZV, Scherer PE (2014). Adipocyte inflammation is essential for healthy adipose tissue expansion and remodeling. Cell Metab.

[40] de Punder K, Pruimboom L (2015). Stress induces endotoxemia and low-grade inflammation by increasing barrier permeability. Front Immunol.

[41] Drucker DJ, Erlich P, Asa SL, Brubaker PL (1996). Induction of intestinal epithelial proliferation by glucagon-like peptide 2. Proc Natl Acad Sci U S A.

[42] Pandiri AR (2013). Overview of exocrine pancreatic pathobiology. Toxicol Pathol.

[43] Meek CL, Lewis HB, Reimann F, Gribble FM, Park AJ (2016). The effect of bariatric surgery on gastrointestinal and pancreatic peptide hormones. Peptides..

[44] Rowlands J, Heng J, Newsholme P, Carlessi R (2018). Pleiotropic effects of GLP-1 and analogs on cell signaling, metabolism, and function. Front Endocrinol (Lausanne).

[45] Essah PA, Nestler JE, Sistrun SN, Kelly SM, Levy JR (2007). Effect of macronutrient composition on postprandial peptide YY levels. J Clin Endocrinol Metab.

[46] Helou N, Obeid O, Azar S, Hwalla N (2008). Variation of postprandial PYY3–36Response following ingestion of differing macronutrient meals in obese females. Ann Nutr Metab.

[47] Carr RD, Larsen MO, Winzell MS, Jelic K, Lindgren O, Deacon CF, Ahrén B (2008). Incretin and islet hormonal responses to fat and protein ingestion in healthy men. Am J Physiol Endocrinol Metab.

[48] van der Klaauw AA, Keogh JM, Henning E, Trowse VM, Dhillo WS, Ghatei MA, Farooqi IS (2013). High protein intake stimulates postprandial GLP1 and PYY release. Obesity (Silver Spring).

[49] Adams SH, Lei C, Jodka CM, Nikoulina SE, Hoyt JA, Gedulin B, Mack CM, Kendall ES (2006). PYY[3-36] administration decreases the respiratory quotient and reduces adiposity in diet-induced obese mice. J Nutr.

[50] Guo Y, Ma L, Enriori PJ, Koska J, Franks PW, Brookshire T, Cowley MA, Salbe AD, Delparigi A, Tataranni PA (2006). Physiological evidence for the involvement of peptide YY in the regulation of energy homeostasis in humans. Obesity (Silver Spring).

[51] Chang SW, Pan WS, Lozano Beltran D, Oleyda Baldelomar L, Solano MA, Tuero I, Friedland JS, Torrico F, Gilman RH (2013). Gut hormones, appetite suppression and cachexia in patients with pulmonary TB. PLoS One.

[52] Grandt D, Schimiczek M, Beglinger C, Layer P, Goebell H, Eysselein VE, Reeve JR Jr (1994). Two molecular forms of peptide YY (PYY) are abundant in human blood: characterization of a radioimmunoassay recognizing PYY 1–36 and PYY 3–36. Regul Pept.

[53] Chelikani PK, Haver AC, Reidelberger RD (2005). Intravenous infusion of peptide YY(3–36) potently inhibits food intake in rats. Endocrinology..

[54] van Avesaat M, Troost FJ, Ripken D, Hendriks HF, Masclee AAM (2015). Ileal brake activation: macronutrient-specific effects on eating behavior?. Int J Obes.

[55] Anderson GH, Moore SE (2004). Dietary proteins in the regulation of food intake and bodyweight in humans. J Nutr.

[56] Shin HS, Ingram JR, McGill A-T, Poppitt SD (2013). Lipids, CHOs, proteins: can all macronutrients put a ‘brake’ on eating*?*. Physiol Behav.

[57] Pezeshki A, Fahim A, Chelikani PK (2015). Dietary whey and casein differentially affect energy balance, gut hormones, glucose metabolism, and taste preference in diet-induced obese rats. J Nutr.

[58] Stengel A, Goebel-Stengel M, Wang L, Hu E, Karasawa H, Pisegna JR (2013). High-protein diet selectively reduces fat mass and improves glucose tolerance in Western-type diet-induced obese rats. Am J Phys Regul Integr Comp Phys.

[59] Fromentin G, Darcel N, Chaumontet C, Marsset-Baglieri A, Nadkarni N, Tomé D (2012). Peripheral and central mechanisms involved in the control of food intake by dietary amino acids and proteins. Nutr Res Rev.

[60] Tomé D, Darcel N, Faipoux R, Gougis S, Fromentin G (2008). Proteins activate satiety-related neuronal pathways in the brainstem and hypothalamus of rats. J Nutr.

[61] L'Heureux-Bouron D, Tomé D, Bensaid A, Morens C, Gaudichon C, Fromentin G (2004). A very high 70%-protein diet does not induce conditioned taste aversion in rats. J Nutr.

[62] Swenson BR, Saalwachter Schulman A, Edwards MJ, Gross MP, Hedrick TL, Weltman AL (2007). The effect of a low-carbohydrate, high-protein diet on post laparoscopic gastric bypass weight loss: a prospective randomized trial. J Surg Res.

[63] la Fleur SE, Luijendijk MCM, van der Zwaal EM, Brans MAD, Adan RAH (2014). The snacking rat as model of human obesity: effects of a free-choice high-fat high-sugar diet on meal patterns. Int J Obes.

[64] Pan A, Hu FB (2011). Effects of carbohydrates on satiety: differences between liquid and solid food. Curr Opin Clin Nutr Metab Care.

[65] Apolzan JW, Harris RB (2012). Differential effects of chow and purified diet on the consumption of sucrose solution and lard and the development of obesity. Physiol Behav.

[66] Ackroff K, Bonacchi K, Magee M, Yiin Y-M, Graves JV, Sclafani A (2007). Obesity by choice revisited: effects of food availability, flavor variety and nutrient composition on energy intake. Physiol Behav.

[67] Gomez-Smith M, Karthikeyan S, Jeffers MS, Janik R, Thomason LA, Stefanovic B, Corbett D (2016). A physiological characterization of the cafeteria diet model of metabolic syndrome in the rat. Physiol Behav.

[68] Mikuska L, Vrabcová M, Lackovicova L, Ukropec J, Hegedusova N, Slavkovsky P (2013). Long-term liquid nutrition intake and development of obesity: differences between young and adult rats. Endocr Regul.

[69] Zheng M, Allman-Farinelli M, Heitmann BL, Toelle B, Marks G, Cowell C (2014). Liquid versus solid energy intake in relation to body composition among Australian children. J Hum Nutr Diet.

[70] Stull AJ, Apolzan JW, Thalacker-Mercer AE, Iglay HB, Campbell WW (2008). Liquid and solid meal replacement products differentially affect postprandial appetite and food intake in older adults. J Am Diet Assoc.

[71] Cassady BA, Considine RV, Mattes RD (2012). Beverage consumption, appetite, and energy intake: what did you expect?. Am J Clin Nutr.

[72] Rayner DV, Corneloup J, Moar KM, Barrett P, Archer ZA, Mercer JG (2007). Solid and liquid obesogenic diets induce obesity and counter-regulatory changes in hypothalamic gene expression in juvenile Sprague-Dawley rats. J Nutr.

